# Matrons and Nurses Who Prefer the "London" Training

**Published:** 1918-08-17

**Authors:** E. Margaret Cummins

**Affiliations:** Liverpool Royal Infirmary


### THE EDITOR'S LETTER-BOX.

### Matrons and Nurses who Prefer the "London" Training.

*To the Editor of* THE HOSPITAL.

SIR,—The London Hospital has been more than usual in the public eye lately, and I venture no opinion on the two, three, or four years' training controversy. But *as* a "Londoner" who was fortunate to spend her three years in hospital, I frequently meet a number of matrons who also trained there, not one of whom have I heard definitely express any other remark than that she would train there again were she beginning her career, which really sums up the spirit of *esprit de corps* and is the salt of life in any and every good training-school, and is what I have to-day been endeavouring to impress upon my own nurses in my lecture.—I am yours faithfully, E. MARGARET CUMMINS.

Liverpool Royal Infirmary,

August 10, 1918.

